# Growth of Novel Ceramic Layers on Metals via Chemical and Heat Treatments for Inducing Various Biological Functions

**DOI:** 10.3389/fbioe.2015.00176

**Published:** 2015-10-27

**Authors:** Tadashi Kokubo, Seiji Yamaguchi

**Affiliations:** ^1^Department of Biomedical Sciences, College of Life and Health Sciences, Chubu University, Kasugai, Japan

**Keywords:** bioactivity, osteoconduction, osteoinduction, Ti metal, Ti-based alloys, apatite, alkali and heat treatment, acid and heat treatment

## Abstract

The present authors’ systematic studies on growth of novel ceramic layers on Ti metal and its alloys by chemical and heat treatments for inducing bone-bonding bioactivity and some other biological functions are reviewed. Ti metal formed an apatite on its surface in a simulated body fluid, when heat-treated after exposure to strong acid solutions to form rutile surface layer, or to strong alkali solutions to form sodium titanate surface layer. Both types of Ti metal tightly bonded to the living bone. The alkali and heat treatment was applied to the surface Ti metal of an artificial hip joint and successfully used in the clinic since 2007. The acid and heat treatments was applied to porous Ti metal to induce osteoconductivity as well as osteoinductivity. The resulting product was successfully used in clinical trials for spinal fusion devices. For the Ti-based alloys, the alkali and heat treatment was little modified to form calcium titanate surface layer. Bone-growth promoting Mg, Sr, and Zn ions as well as the antibacterial Ag ion were successfully incorporated into the calcium titanate layer.

## Introduction

A considerable amount of material is needed to repair bone defects in both orthopedics and dentistry. Autogenous bone is the best bone substitute material in terms of compatibility with the surrounding tissue. However, only a small amount of bone can be harvested from the healthy part. Allogenic bone is the second choice, commonly results in side effects such as foreign body reaction and/or infection. Therefore, synthetic materials free of antigens or toxic impurities are obviously required.

Various kinds of synthetic materials have been used as bone substitutes, including organic polymers, ceramics, metals, and composites. However, most of them are encapsulated in a collagenous fibrous tissue so as to be isolated from living bone (Park and Lakes, 1992). This is a normal foreign body reaction that protects living tissue. However, because of this reaction, synthetic materials do not become stably fixed to the surrounding bone for a long period of time.

In contrast, certain kinds of ceramics such as Bioglass^®^ in Na_2_O–CaO–SiO_2_–P_2_O_5_, glass-ceramic A-W-containing crystalline apatite and Wollastonite in MgO–CaO–SiO_2_–P_2_O_5_, sintered hydroxyapatite of the composition Ca_10_(PO_4_)_6_(OH)_2_, sintered β-tricalcium phosphate of the composition 3CaO⋅P_2_O_5_, and biphasic calcium phosphates composed of hydroxyapatite and β-tricalcium phosphate have all been found to bond to living bone without forming fibrous tissue around them. They are called “bioactive ceramics” and are already in clinical uses as important bone substitutes (Kokubo, 2008). However, their mechanical strength and fracture toughness are not as high as those of human cortical bone, so they cannot be used under load-bearing conditions.

Under load-bearing conditions, metallic materials such as stainless steel, Co–Cr–Mo alloys, titanium (Ti) metal, and its alloys are mainly used, because of their high mechanical strengths and superior corrosion resistances (Park and Lakes, 1992). However, even Ti metal and Ti-based alloys, which exhibit best biocompatibility among them, are also encapsulated by fibrous tissue and do not bond to living bone (Hacking et al., 2002). Their fixation also does not become stable for long period of time.

In order to confer bone-bonding bioactivity to the metallic materials, some bioactive ceramics such as hydroxyapatite have been coated on them using various techniques, such as plasma spraying, flame spraying, sputtering, sol–gel deposition, biomimetic method (Leeuwenburgh et al., 2008), and alternating soaking (Taguchi et al., 2001). However, the coated layer is not stable in the living body, because of its vulnerability to cracking, transformation, and degradation (Leeuwenburgh et al., 2008).

The present authors recently reported that certain metallic materials such as Ti metal and its alloys exhibit bone-bonding bioactivity when a certain kind of thin ceramic layer is grown on their surface via simple chemical and heat treatments. In the present paper, the recent results of our research on this subject are reviewed.

## Basic Concept for Conferring Bone-Bonding Bioactivity to Metals

It was found by the present authors that Bioglass^®^, glass-ceramic A-W and sintered hydroxyapatite bond to living bone through an apatite layer that forms on their surfaces in the living body. In contrast, glass-ceramic A-W(Al) containing crystalline apatite and wollastonite similar to glass-ceramic A-W, but added with a small amount of Al_2_O_3_, neither formed the apatite on its surface in the living body nor bonded to living bone. Consequetly, it was concluded that a material able to form the apatite on its surface in the living body is able to bond to living bone through the apatite layer, but a material unable to form the surface apatite does not bond to living bone (Kokubo and Takadama, 2006). Therefore, it is expected that even metallic materials will bond to living bone when their surfaces are modified such that they form the apatite on their surface *in vivo*.

Is it necessary that every metal subjected to different surface modifications should be implanted into animal bone defects to check for the apatite formation on their surfaces? This would require not only considerable cost and time but also the sacrifice of a great many animals.

We demonstrated that the apatite formation on the surfaces of Bioglass^®^, glass-ceramic A-W and sintered hydroxyapatite *in vivo* can be reproduced, even in an acellular simulated body fluid (SBF) having ion concentrations almost equal to those of the human blood plasma. In contrast, glass-ceramic A-W(Al) did not form apatite on its surface in SBF nor bonded in the living body. This means that the apatite formation on a material in the living body can be evaluated in SBF without the need of animal experiments (Kokubo and Takadama, 2006).

The next problem was to determine the kind of material that effectively induces apatite formation in SBF. We found that certain simple metal oxide gels, such as TiO_2_, ZrO_2_, Nb_2_O_5_, and Ta_2_O_5_ form the apatite on their surfaces in SBF within a week (Li et al., 1994). Metallic materials are generally covered with a thin oxide layer. In view of this fact, it is expected that metallic materials based on Ti, Zr, Nb, and Ta form the apatite on their surfaces in SBF, as well as in the living body, so as to be able to bond to living bone when their surfaces are appropriately modified.

## Surface Modification of Metals

Ti metal and its alloys are the most widely used type of material as implants in the orthopedic and dental fields among the metallic materials described above because of their better compatibility with living tissue. Therefore, various surface modifications were applied to Ti metal and its alloys for inducing bone-bonding bioactivity using various methods, including ion implantation (Armitage et al., 2007; Nayab et al., 2007; Rautray et al., 2010), electrochemical reaction (Suh, 2003; Bjursten et al., 2010; Shibata et al., 2010; Whiteside et al., 2010; Zhao et al., 2010; Diefenback et al., 2011; Xie et al., 2011; Minagar et al., 2013; Zhang et al., 2013; Zhou et al., 2014), and hydrothermal treatments (Dong et al., 2007; Park et al., 2007, 2010; Chen et al., 2009; Ueda et al., 2009; Zhang et al., 2010). However, these techniques require special equipment and are not readily applicable to large-scale medical devices of complicated or porous structure. In contrast with these techniques, chemical and heat treatments do not have such limitations.

Various kinds of chemical and heat treatments also have been applied to Ti metal and its alloys to induce apatite formation on their surfaces in SBF (Wang et al., 2002; Wu et al., 2004; Takeuchi et al., 2005; Zhao et al., 2005, 2008; Cooper et al., 2006; Lee et al., 2007; Liu et al., 2007; Zhou et al., 2007; Sugino et al., 2009; Karthega and Rajendran, 2010; Li et al., 2010; Turkan and Guden, 2010; Ferraris et al., 2011). However, few results in these studies have been considered in terms of the mechanism of the apatite formation and correlated with the *in vivo* bone-bonding bioactivity.

The following are systematic studies we conducted on chemical and heat treatments of Ti metal and its alloys for inducing apatite formation on their surfaces in SBF along with their correlation with *in vivo* bone-bonding bioactivity.

## Simple Chemical and Heat Treatments of Ti Metal

**Figure 1:**
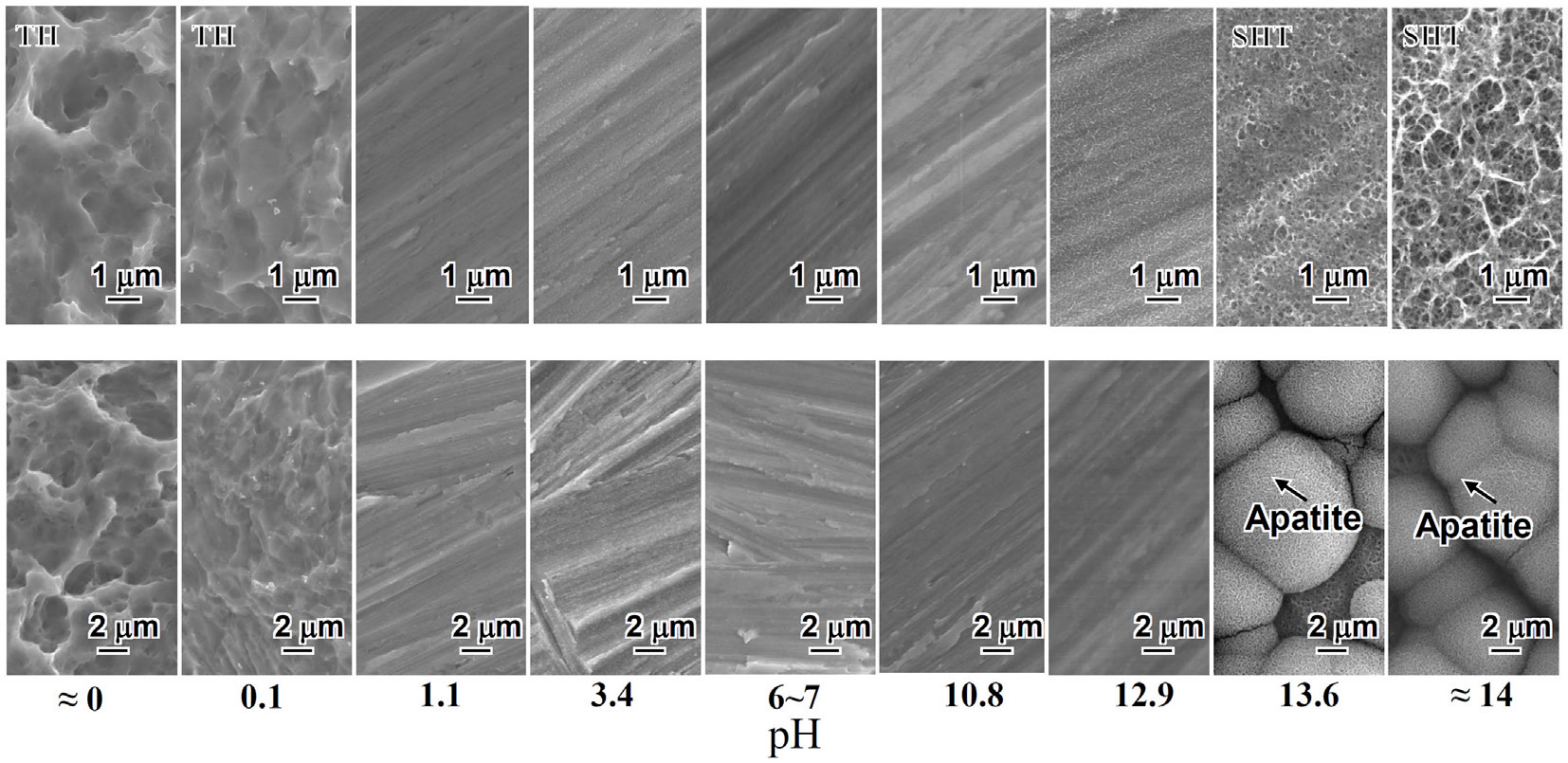
**SEM photographs of surfaces of Ti metal exposed to solutions with different pHs (top) and those of the same surfaces after soaking in SBF for 3 days (bottom)**. TH, titanium hydride; SHT, sodium hydrogen titanate. Reproduced from Pattanayak et al. (2012) with permission The Royal Society.

When a rectangular plate of Ti metal was soaked in an aqueous solution in which the pH was systematically changed from almost 0 to 14 by HCl or NaOH at 60°C for 24 h, it formed micrometer-scale roughness, precipitating titanium hydride (TiH_x_) on its surface for the strong acid solutions <1.1 in pH, and nanometer-scale roughness precipitating sodium hydrogen titanate (Na_x_H_2−x_Ti_3_O_7_, 0 < *x* < 2) on its surface (Yamaguchi et al., 2009) for the strong alkali solutions higher than 13.6 in pH, as shown in Figure 1.

Only the Ti metal soaked in the strong alkali solutions higher than 13.6 in pH formed the apatite on its surface in SBF within 3 days, as shown in Figure 1 (Pattanayak et al., 2012).

**Figure 2:**
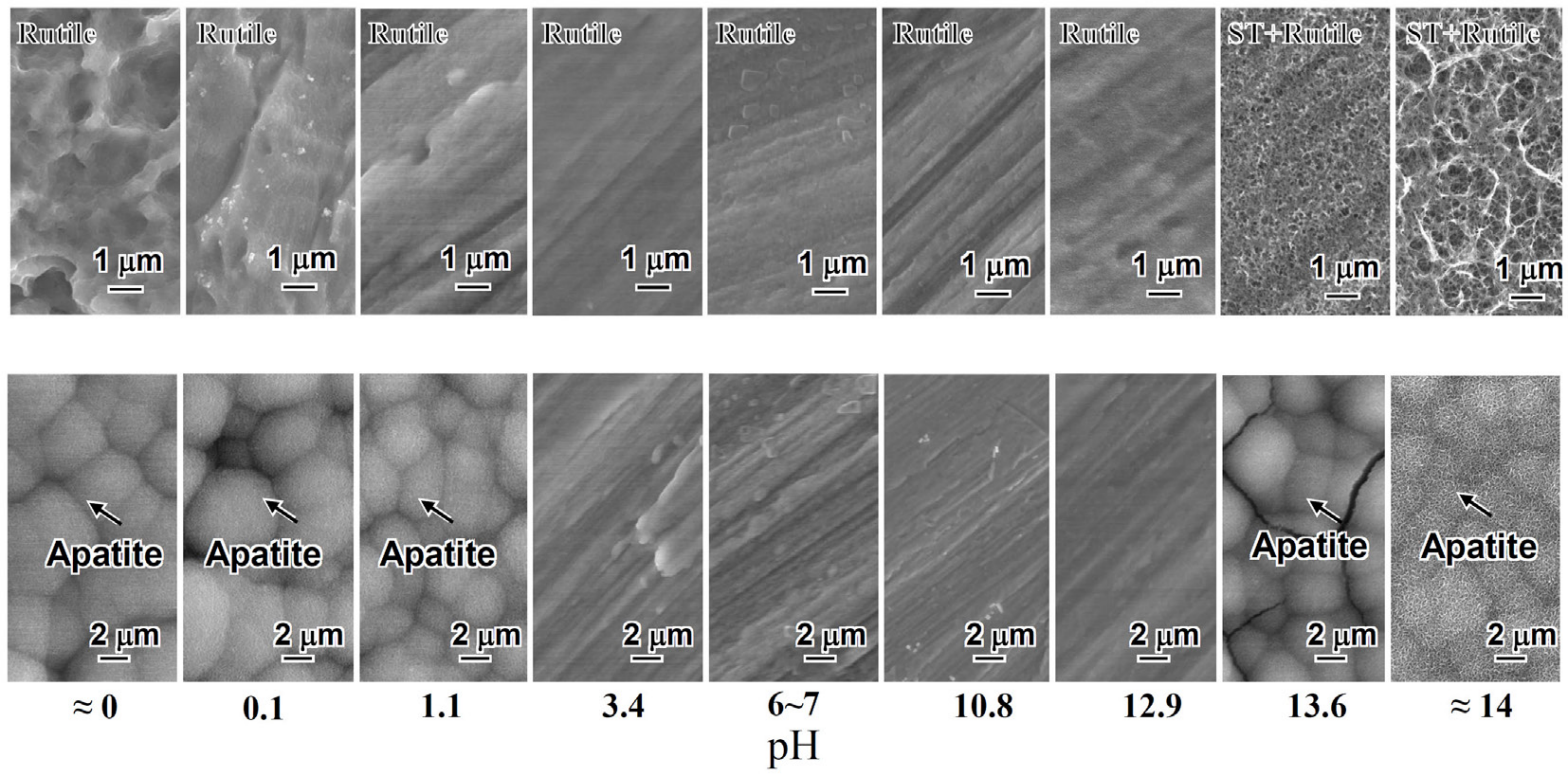
**SEM photographs of surfaces of Ti metal heat-treated after exposure to solutions with different pHs (top) and those of the same surfaces after soaking in SBF (bottom)**. ST, sodium titanate. Reproduced from Pattanayak et al. (2012) with permission The Royal Society.

When the Ti metal was heat-treated at 600°C for 1 h after exposure to the solutions described above, no surface morphological change was observed on its surface as the result of the heat treatment. However, the titanium hydride was transformed into rutile (TiO_2_), while the sodium hydrogen titanate was transformed into sodium titanate (Na_2_Ti_6_O_13_) and rutile by the heat treatment. The Ti metals exposed to the solutions with intermediate pH values also precipitated the rutile on their surfaces by the heat treatment, as shown in Figure 2.

Among them, only the Ti metals heat-treated after exposure to the strong acid solutions <1.1 in pH or strong alkali solutions higher than 13.6 in pH formed the apatite on their surfaces in SBF within 3 days, as shown in Figure 2 (Pattanayak et al., 2012).

**Figure 3:**
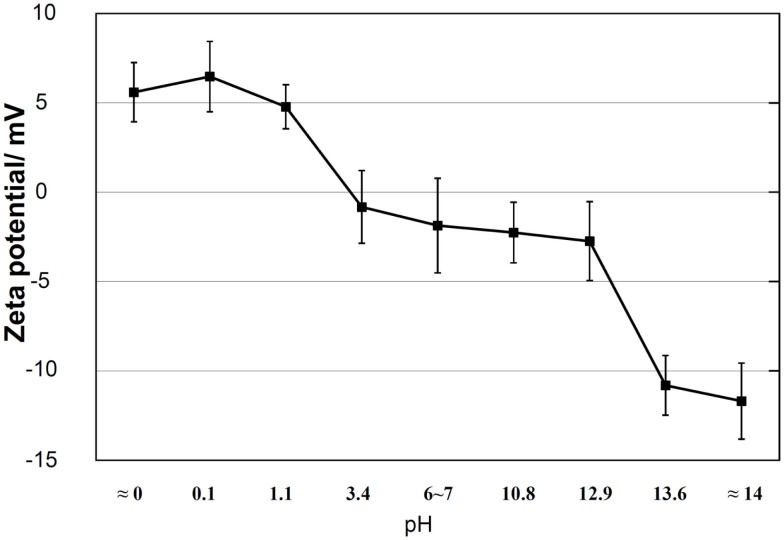
**Zeta potentials of Ti metal heat-treated after exposure to solutions with different pH values**. Reproduced from Pattanayak et al. (2012) with permission The Royal Society.

It is clear from these results that the apatite formation on Ti metal depends upon neither the specific surface roughness nor crystalline phase. When the zeta potential of the Ti metal heat-treated after exposure to the solutions is measured, it can be seen from Figure 3 that the apatite formation on the Ti metal is induced by the positive or negative surface charge (Pattanayak et al., 2012).

**Figure 4:**
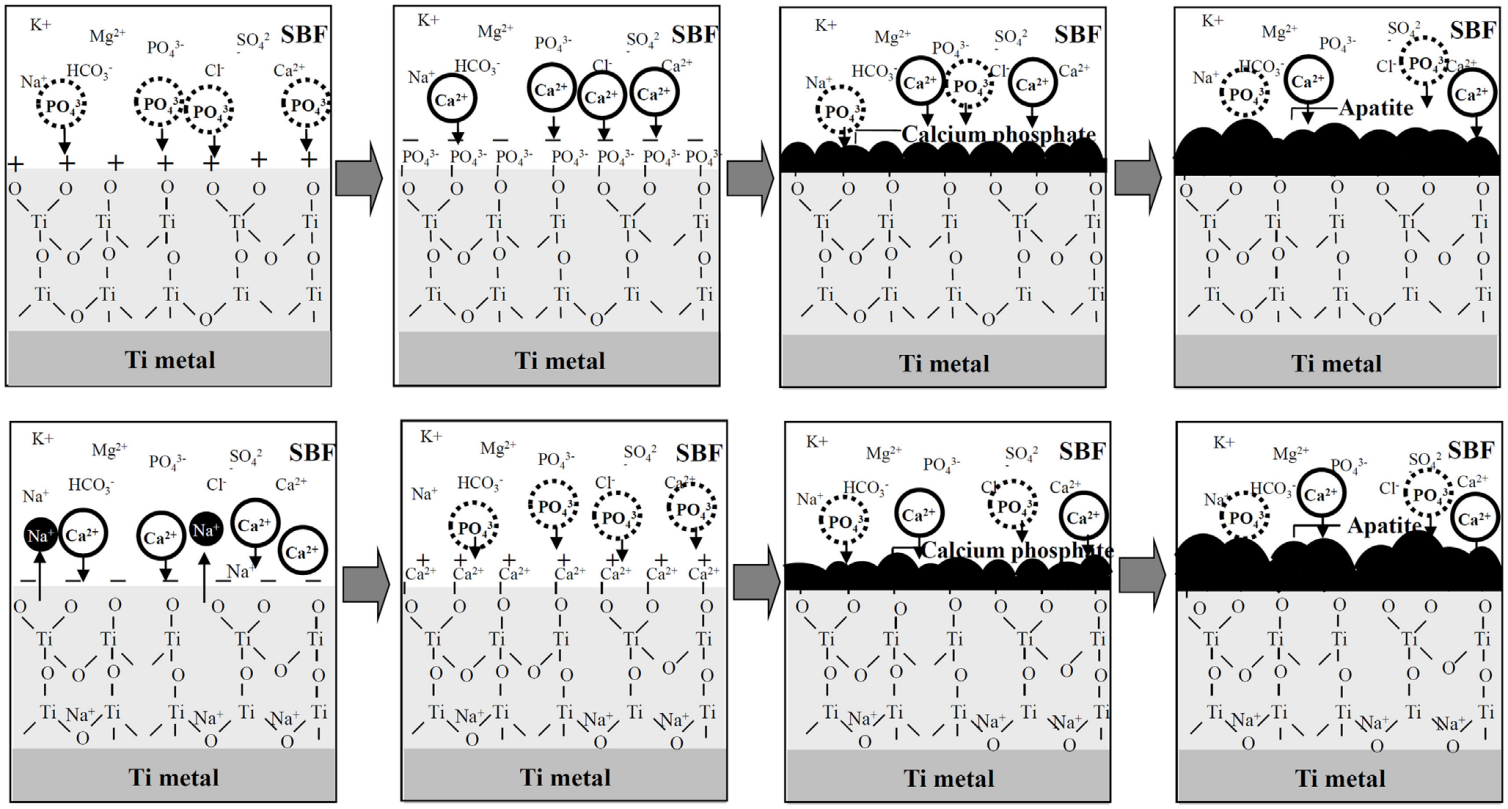
**Process of apatite formation on Ti metals heat-treated after exposure to strong acid solutions (top), and to strong alkali solutions (bottom)**. Reproduced from Pattanayak et al. (2012) with permission The Royal Society.

A positively charged surface might preferentially adsorb negatively charged phosphate ions in SBF. As the phosphate ions accumulate, its surface becomes negatively charged so as to combine with the positively charged calcium ions, forming an amorphous calcium phosphate. This calcium phosphate is metastable and eventually transforms into stable crystalline apatite, as shown in Figure 4 (Pattanayak et al., 2012).

In contrast, a negatively charged surface would be expected to first preferentially adsorb positively charged calcium ions. As the calcium ions accumulate, its surface becomes positively charged so as to combine with the negatively charged phosphate ions, forming an amorphous calcium phosphate and then the crystalline apatite (Kim et al., 2003).

This sequential adsorption of the phosphate and calcium ions was confirmed by X-ray photoelectron spectroscopy (XPS) (Takadama et al., 2001a) and transmission electron microscopic observation combined with energy dispersive X-ray analysis (Takadama et al., 2001b) of the surface of the Ti metal soaked in SBF for different periods of time, as well as for the Ti metal heat-treated after exposure to the strong acid or alkali solution (Pattanayak et al., 2012).

The positive surface charge of the Ti metal heat-treated after exposure to the strong acid solution might be explained as follows. The Ti metal becomes adsorbed with Cl^−^ ions during the acid treatment. The Cl^−^ ions remain even after the heat treatment, and are dissociated in SBF so as to produce a local acidic environment on the Ti metal. It has been reported that titanium oxide is positively charged in an acidic environment (Gold et al., 1989). Consequently, the surface of the Ti metal heat-treated after exposure to strong acid solution is positively charged.

The negative surface charge of the Ti metal heat-treated after exposure to the strong alkali solutions might be explained as follows. The sodium titanate on the surface of the Ti metal releases Na^+^ ions via exchange with the H_3_O^+^ ions in SBF so as to produce a local alkaline environment on the Ti metal. It has been reported that titanium oxide is negatively charged in an alkaline environment (Gold et al., 1989). Consequently, the surface of the Ti metal heat-treated after exposure to the alkali solution is negatively charged.

When the Ti metal is heat-treated after exposure to a neutral solution, it is not charged, since no charge is produced on its surface. When it is not heat-treated after exposure to the solution, it is not charged except after exposure to the strong alkali solutions, since an electrically insulating oxide layer is not produced on its surface except in the last case. As result, the apatite is not formed on the Ti metal surface in SBF in these cases except the last, as may be seen in Figures 1 and 2 (Pattanayak et al., 2012).

This dependence of the apatite formation on the Ti metal upon the pH of the exposed solution is valid for different kinds of acid and alkali solutions. For example, the Ti metal also forms the apatite on its surface in SBF when heat-treated at 600°C for 1 h after exposure to strong 66.3%H_2_SO_4_/10.6%HCl acid solution at 70°C for 24 h (Kokubo et al., 2010).

**Figure 5:**
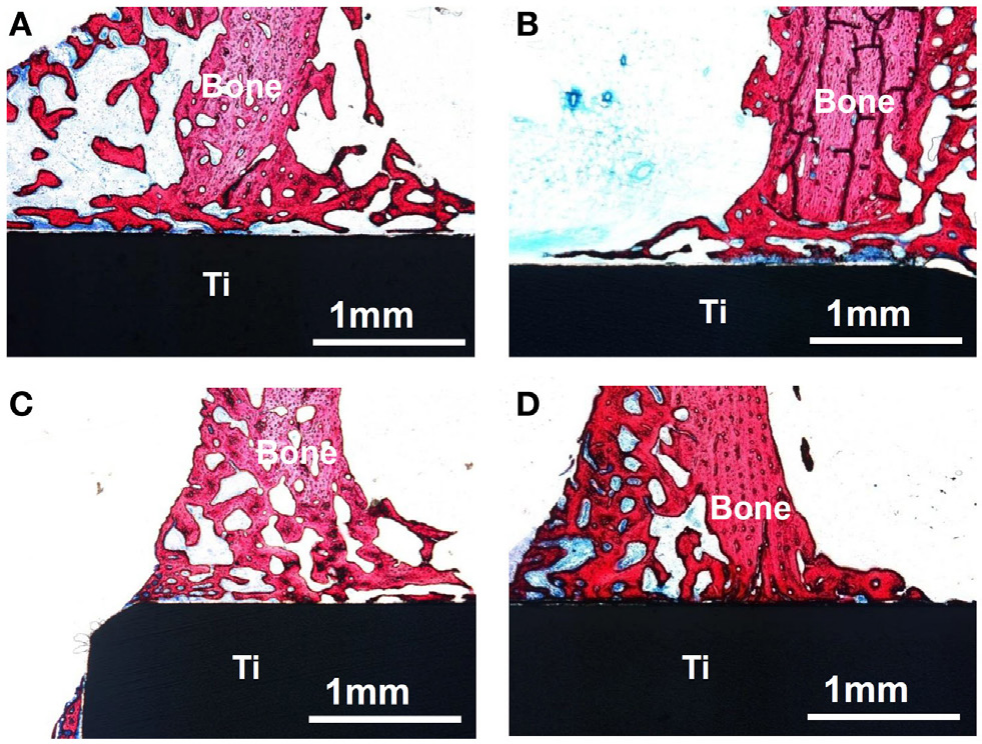
**Optical micrographs of non-decalcified sections of Ti metals subjected to no treatment (A), only H_2_SO_4_/HCl acid treatment (B), only heat treatment at 600°C (C) and H_2_SO_4_/HCl acid and heat treatment at 600°C (D), 4 weeks after implantation into the tibia of rabbit**. Reproduced from Kawai et al. (2012) with permission Springer.

When the Ti metal subjected to the H_2_SO_4_/HCl acid and heat treatments was implanted into a rabbit tibia, it came into direct contact with the surrounding bone within 4 weeks, without any intervening fibrous tissue at the interface, as shown in Figure 5D. The metal became so tightly bonded to the bone that fracture did not occur at their interface but rather in the bone itself when tensile stress was applied to the interface. In contrast, Ti metals subjected to no treatment, only to the H_2_SO_4_/HCl acid treatment or only to the heat treatment at 600°C, were encapsulated by fibrous tissue, as shown in Figures 5A–C (Kawai et al., 2012).

**Figure 6:**
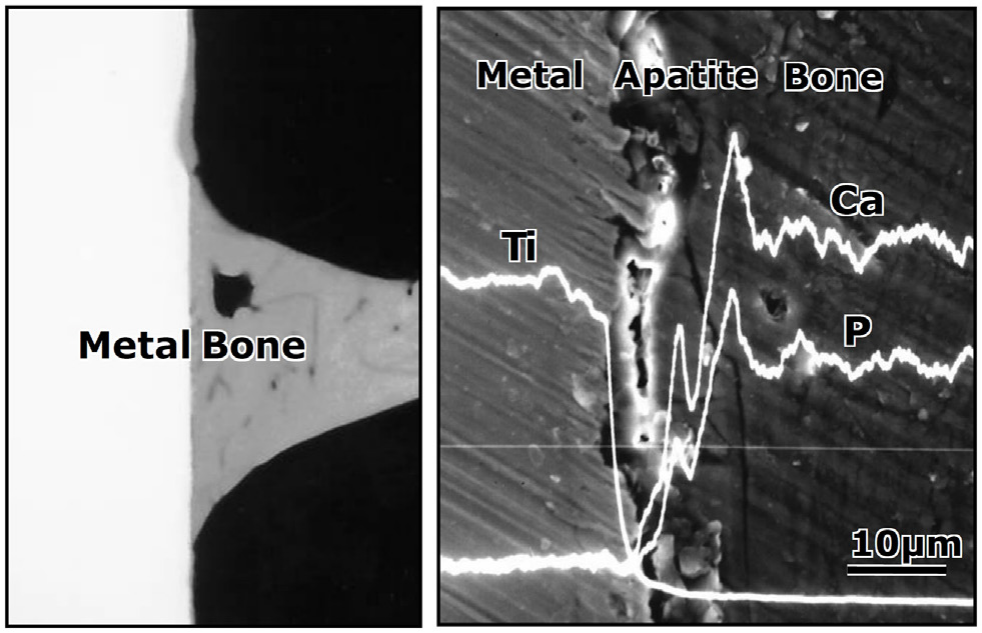
**Contact radiomicrograph (left hand) and SEM photograph (right hand) of Ti metal heat-treated after exposure to NaOH solution at its interface with the bone, 8 weeks after implantation into the tibia of rabbits**. Reproduced from Yan et al. (1997) with permission John Willey and Sons.

**Figure 7:**
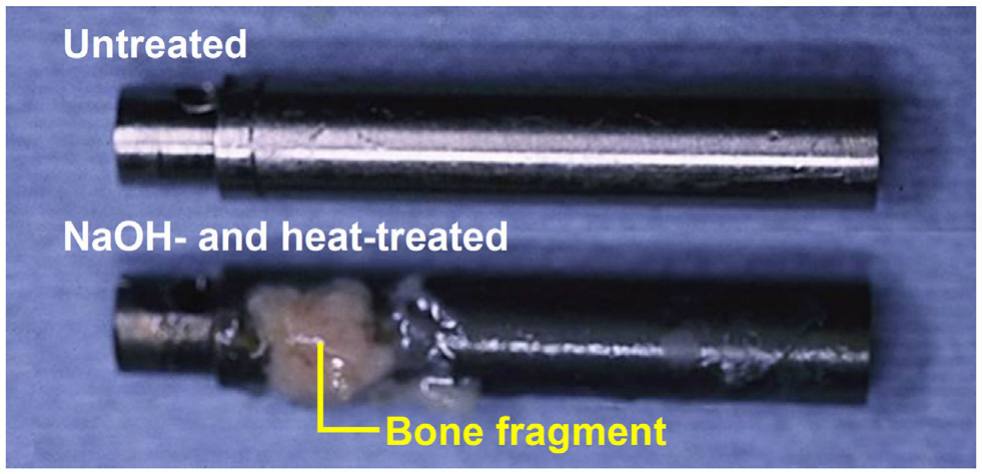
**Titanium metal rod untreated (top) and heat-treated after exposure to NaOH solution (bottom), both of which were pulled out after implanted into medullary canal of a rabbit for 12 weeks, after Nishiguchi et al. (2003)**.

When the Ti metal plate heat-treated at 600°C after exposure to 5M NaOH solution at 60°C for 24 h was implanted into a rabbit tibia, within 8 weeks it became bonded to the surrounding bone through an apatite layer that formed on the Ti metal surface, as shown in Figure 6 (Yan et al., 1997). When a rod of the same metal was implanted into the medullary canal of the rabbit femur, it became so tightly bonded to the surrounding bone within 12 weeks that it was not able to be extracted without accompanying bone fragments, as shown in Figure 7 (Nishiguchi et al., 2003).

These *in vivo* bone-bonding bioactivity findings of the Ti metals are consistent with the *in vitro* apatite formation in SBF of the Ti metals. This consistency could be interpreted in terms of cell response as follows. The Ti metal able to form the apatite on its surface in SBF forms the apatite on its surface soon after implanted into living body. Once the apatite is formed, osteogenic cells actively differentiate on its surface, as observed in culture experiments of rat bone marrow cells (Nishio et al., 2000) and of mouse calvaria osteoblast (Isaac et al., 2010) on a NaOH- and heat-treated Ti metal. Once the bone matrix is formed by the osteogenic cells, it can be tightly bonded to the apatite on the surface of the Ti metal, as observed for bone nodules, which was produced by cells migrated from the calvaria explant of rat, on a NaOH- and heat-treated Ti metal (Isaac et al., 2009). Recently, active proliferation and differentiation of osteoblast and multifocal nodule formation on the NaOH- and heat-treated Ti metal were observed for the Ti metal implanted into mouse by using fluorescent osteoblast *in vivo* (Tsukanaga et al., 2013).

It is apparent from these results that the natural Ti metal is unable to bond to living bone. When it is subjected to only the acid treatment or only the heat treatment, it also cannot bond to living bone (Kawai et al., 2012). However, it does bond to living bone, when it is heat-treated after exposure to strong acid or alkali solution so that a certain kind of thin ceramic layer is grown on its surface.

## Clinical Applications of the Bone-Bonding Bioactive Ti Meal Prepared by Simple Chemical and Heat Treatments

It is well known that commercial dental implants may be subjected to H_2_SO_4_/HCl acid solution treatment in order to produce micrometer-scale roughness on their surface (Coelho et al., 2009). This treatment can promote mechanical interlocking of the dental implants with the surrounding bone. However, this treatment cannot make direct bonding of the dental implants to the surrounding bone.

The present result indicates that such implants can be induced to bond to bone when subjected to heat treatment after acid treatment, since a rutile layer capable of forming apatite in the living body is formed on its surface by the heat treatment. Such dental implants are expected to remain stably fixed over a long period of time after implantation.

**Figure 8:**
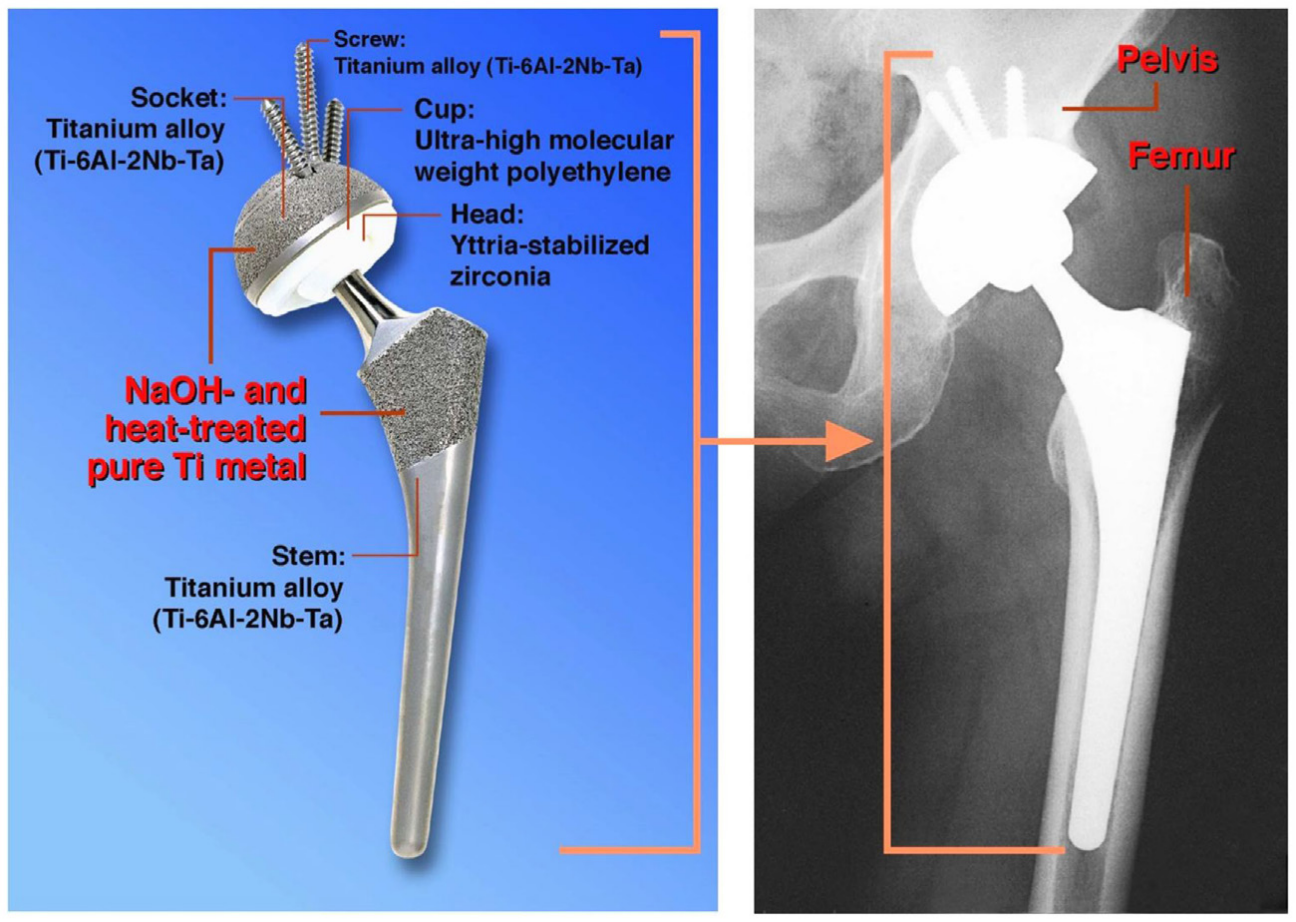
**Artificial hip joint, porous Ti metal layer of which was heat-treated after exposure to NaOH solution, after Kawanae et al. (2009)**.

It is expected that once a porous Ti metal layer has been produced on the surface of a Ti-based alloy, for example by a plasma spray method, and then subjected to the NaOH and heat treatments, such a medical device will be stably fixed to the surrounding bone, since the surrounding bone comes into direct contact with the porous Ti metal and grows into the pores (Kim et al., 2000c; Nishiguchi et al., 2001). Such a bone-bonding bioactive porous Ti metal layer was produced on the surface of accetabular shell and femoral stem of a total artificial hip joint made of Ti–6Al–2Nb–Ta alloy, as shown in Figure 8. This hip joint has been clinically used in more than 15,000 patients in Japan since 2007 (Kawanae et al., 2009). The result was confirmed by two implants retrieved 2 weeks and 8 years after implantation, respectively, due to femoral fracture and infection that the implant surface had become intimately integrated with newly grown bone as early as 2 weeks after implantation and had maintained this integrity for a minimum of 8 years (So et al., 2013).

Currently, hydroxyapatite is commonly coated on commercial artificial hip joints. For example, hydroxyapatite is coated by a flame-spray method on the rough Ti metal layer produced by the arc-spray method on the surface of the Ti–15Mo–5Zr–3Al alloy in one such case. Recently, Kawai et al. tried to apply the NaOH and heat treatments on the surface of the rough Ti metal layer produced on the Ti–5Mo–15Zr–3Al alloy by the arc-spray method for comparison. The rectangular plates subjected to the NaOH and heat treatments and hydroxyapatite coating, as well as that untreated were implanted into the tibia of a rabbit. Consequently, it was found that the bonding strength of the implants to the bone was not increased by the hydroxyapatite coating, but it was considerably increased by the NaOH and heat treatments as early as 4 weeks after implantation, and this increase was maintained even after 16 weeks. The lack of a positive effect of the hydroxyapatite coating was attributed to deterioration of the hydroxyapatite layer in the body (Kawai et al., 2015).

It is expected that this kind of simple chemical and heat treatment will also be useful for inducing bone-bonding bioactivity in different kinds of Ti metal implants in the orthopedic and dental fields. However, it will be ineffective for many kinds of Ti-based alloys. This treatment must be modified for such Ti-based alloys.

## Modification of the Alkali and Heat Treatment

Ti-based alloys such as Ti–6Al–4V, Ti–6Al–2Nb–Ta, and Ti–15Mo–5Zr–3Al exhibit much great mechanical strength and fracture toughness, and hence they are also widely used as implants in orthopedics and dentistry.

The simple heat treatment after exposure to the NaOH solution described above is also effective for these conventional Ti-based alloys in inducing bone-bonding bioactivity (Kim et al., 1996; Ueno et al., 2011). In these cases, alloying elements such as Al, V, and Mo are easily and selectively released during the NaOH treatment, and only the sodium titanate and rutile are precipitated on the surfaces of the alloys after the heat treatment, similar to the pure Ti metal (Kim et al., 1999; Kim et al., 2000a,b). Consequently, they come to exhibit the bone-bonding bioactivity as the result of this simple chemical and heat treatment (Nishiguchi et al., 1999).

On the other hand, Ti-based alloys in the system Ti–Zr–Nb–Ta, which are free of elements suspected of cytotoxicity such as Al and V, were recently developed. Some of these alloys exhibit not only high mechanical strength, but also low elastic modulus close to that of human bone. They are thus expected to be more useful as implants in orthopedics and dentistry. However, the simple alkali and heat treatment is not effective for these alloys in inducing their bone-bonding bioactivity. The alloying elements such as Zr, Nb, and Ta are not as easily released as Al and V during the NaOH treatment, and considerable amounts of them remain even after the subsequent heat treatment, suppressing Na^+^ ion release from the alloys and inhibiting apatite formation in SBF as well as in the body environment (Yamaguchi et al., 2011).

It is well known that Ca^2+^ ion release is more effective for apatite formation than Na^+^ ion release. Therefore, it is expected that even these alloys could form the apatite on their surfaces in SBF as well as in the body environment if the sodium titanate on their surfaces were replaced with calcium titanate. However, calcium titanate cannot be formed by the simple chemical and heat treatment in which the NaOH solution is replaced by Ca(OH)_2_ solution, since the solubility of Ca(OH)_2_ in water is very low.

**Figure 9:**
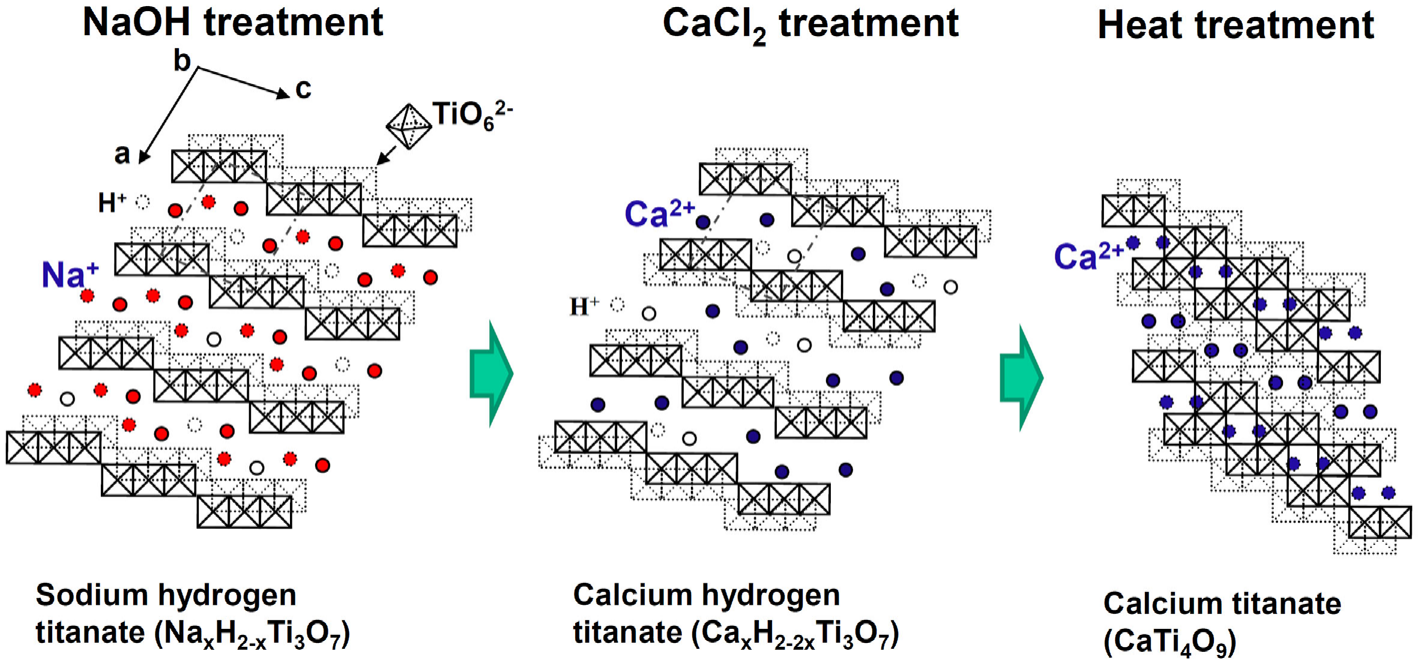
**Structures of sodium hydrogen titanate, calcium hydrogen titanate, and calcium titanate projected on a plane perpendicular to the crystallographic *b* axis**. Reproduced from Kokubo and Yamaguchi (2015) with permission Elsevier.

Fortunately, the sodium hydrogen titanate that forms on the Ti metal and its alloys by NaOH treatment has a well-developed layered structure, as shown in Figure 9 (Kokubo and Yamaguchi, 2015). It is expected that the Na^+^ ions in the layered structure may be completely replaced with the Ca^2+^ ions in the CaCl_2_ solution to form a calcium hydrogen titanate. This phase might be transformed into calcium titanate by the subsequent heat treatment. When the Ti metal and its alloys were heat-treated after exposure to the NaOH and subsequent exposure to 100 mM CaCl_2_ solution at 40°C for 24 h, they formed the calcium titanate on their surfaces, as expected. However, the resulting products did not exhibit apatite formation on their surfaces in SBF. This was attributed to the extremely low release rate of the Ca^2+^ ions from the calcium titanate because of its dense structure (Kizuki et al., 2010).

**Figure 10:**
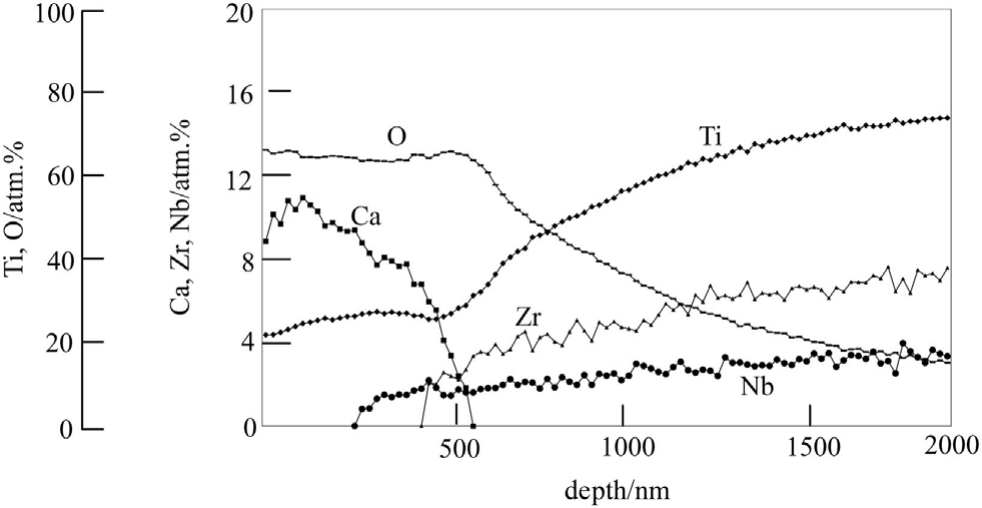
**Depth profile of Auger electron spectroscopy of the surface of Ti–15Zr–4Nb–4Ta alloy subjected to NaOH, CaCl_2_, heat, and water treatments**. Reproduced from Yamaguchi et al. (2010) with permission Springer.

Therefore, they were finally soaked in a hot water, in order to increase the release rate of the Ca^2+^ ions by partly replacing the Ca^2+^ ions at the surface of the calcium titanate with H_3_O^+^ ions in the water, as shown in Figure 10 (Yamaguchi et al., 2010). As a result, the Ti-based alloys in the system Ti–Zr–Nb–Ta, as well as the pure Ti metal, formed the apatite on their surfaces in SBF (Kizuki et al., 2010; Yamaguchi et al., 2010, 2012).

**Figure 11:**
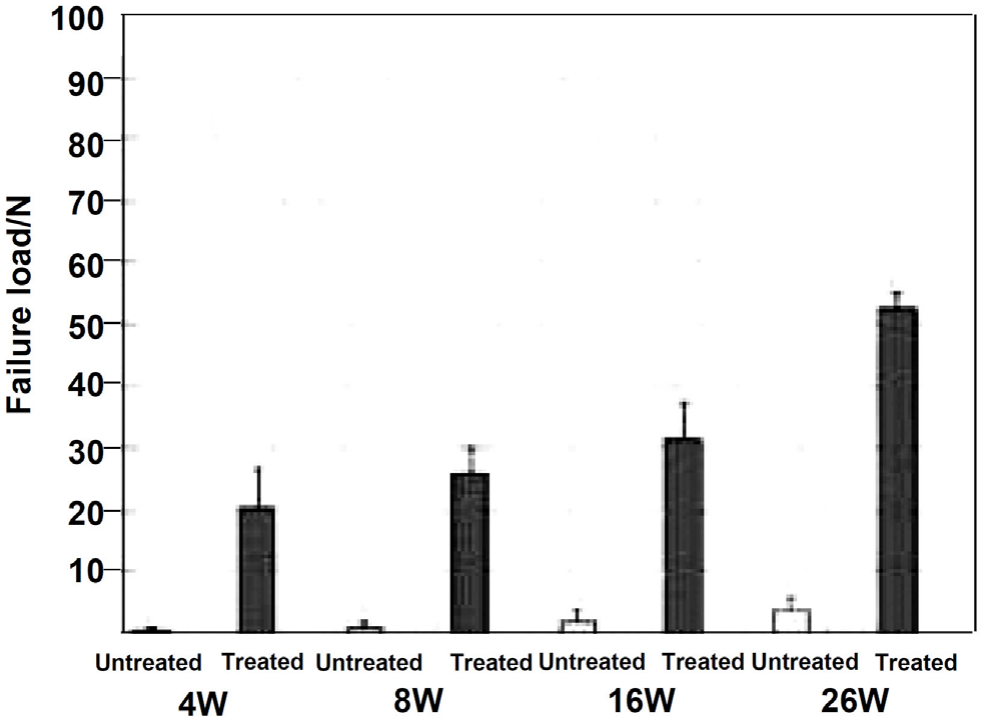
**Failure load under tensile stress perpendicular to the interface of Ti–15Zr–4Nb–4Ta alloy subjected to NaOH, CaCl_2_, heat, and water treatment with the bone, in comparison with that for untreated alloy, after Fukuda et al. (2011a)**.

When the Ti-based alloys as well as the pure Ti metal that had been subjected to this modified alkali and heat treatment for forming the Ca-deficient calcium titanate on their surfaces were implanted into the tibia of a rabbit, they came into direct contact with the surrounding bone without any intervention of fibrous tissue at their interface within 16 weeks (Fukuda et al., 2011a; Tanaka et al., 2014).When a tensile stress was applied to the interface of the Ti–15Zr–4Nb–4Ta alloy with the bone, the load required for producing the fracture was much higher for the alloy subjected to the modified alkali and heat treatment than the untreated alloy, as shown in Figure 11, and this difference increased with an increasing period of time after implantation (Fukuda et al., 2011a).

It should be noted here that this modified alkali and heat treatment that forms Ca-deficient calcium titanate has some advantages over the simple alkali and heat treatment not only for the alloys but also for pure Ti metal, in terms of insensibility to contamination of a NaOH reagent, and humidity in an environment. In general, even so-called high purity NaOH reagent contains small amount of the calcium ions as an impurity. The calcium ions are concentrated on the Ti metal surface during the course of NaOH solution treatment, to give calcium-contaminated sodium titanate after the heat treatment. The incorporated calcium ions suppress apatite formation of the surface of Ti metal in SBF as well as in the living body by inhibiting the Na^+^ ions release, and hence decrease bone-bonding ability (Kizuki et al., 2013).

On the other hand, when the Ti metal and its alloys are stored in a humid environment, the Na^+^ ions in the sodium titanate are liable to be released via exchange with the H_3_O^+^ ions in the moisture, thus decreasing the Na^+^ ions in the sodium titanate decreasing the bone-bonding ability (Kawai et al., 2010). Ti metal and its alloys subjected to the modified alkali and heat treatment so as to form the Ca-deficient calcium titanate on their surfaces have no such problems with regard to these matters.

## Extension of Modified Alkali and Heat Treatment

**Figure 12:**
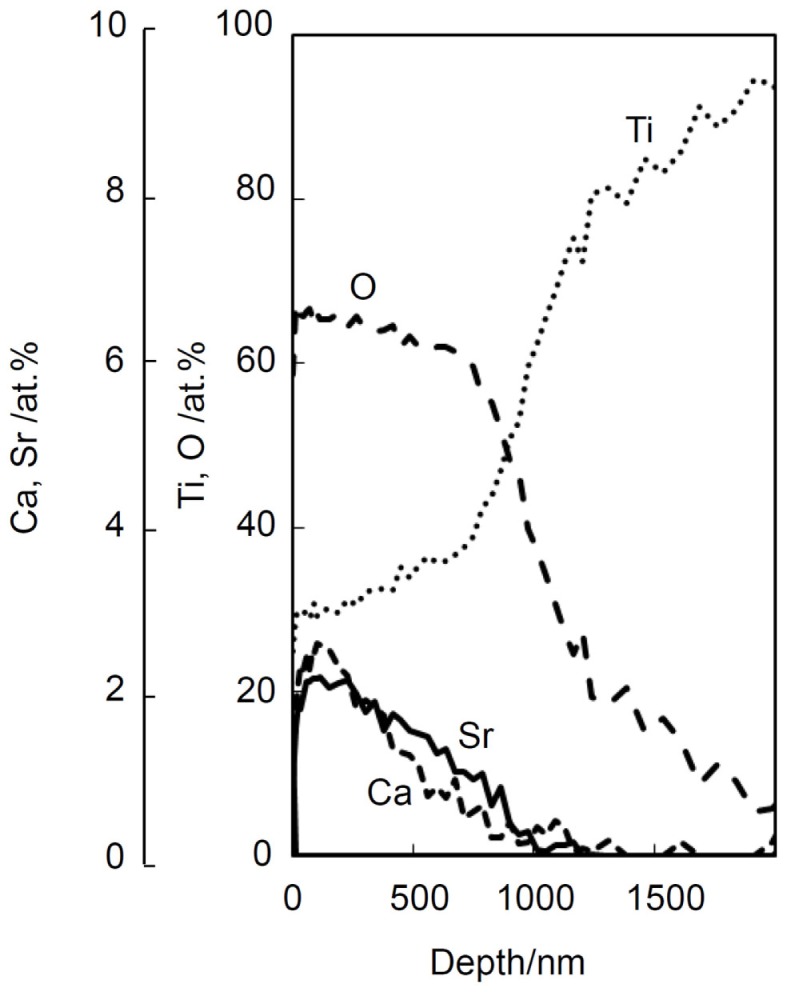
**Depth profile of XPS of Ti metal subjected to NaOH, CaCl_2_/SrCl_2_, heat, and SrCl_2_ treatments**. Reproduced from Yamaguchi et al. (2014) with permission Elsevier.

Certain ions such as Mg (Park et al., 2012), Sr (Bonnelye et al., 2008), and Zn (Alvarez et al., 2010) are known to promote bone growth. It is expected that if these ions are slowly released from the bone-bonding bioactive Ti metal and its alloys, they can be bonded to the surrounding bone in a short period of time after implantation, since the surrounding bone grows to the apatite layer on the surface of the Ti metal in a short period. These ions can be incorporated into the Ca-deficient calcium titanate layer on the surface of the Ti metal and its alloys without disturbing their apatite-forming abilities by adding these ions into the CaCl_2_ solution of the second treatment, and/or into the hot water of the final treatment of the modified alkali and heat treatments (Yamaguchi et al., 2013a,b, 2014). For example, when the Ti metal is heat-treated at 600°C after exposure to a mixed solution of 50 ml CaCl_2_ and 50 ml SrCl_2_ at 40°C for 24 h following to the NaOH solution, and then soaked in 1M SrCl_2_ solution at 80°C for 24 h, the Sr^2+^ ions are incorporated into the Ca-deficient calcium titanate, as shown by depth profile of XPS near the surface of the Ti metal in Figure 12 (Yamaguchi et al., 2014). It was confirmed that this Ti metal can form the apatite on its surface in SBF and that the Sr^2+^ ions are slowly released into the phosphate-buffered saline at 36.5°C (Yamaguchi et al., 2014).

The silver ion, which is known to exhibit antibacterial activity (Chen et al., 2008), also can be incorporated into the Ca-deficient calcium titanate layer of the Ti metal by replacing the final hot water of the modified alkali and heat treatment with 1M AgNO_3_ solution (Kizuki et al., 2014). It was confirmed that this Ti metal also forms the apatite on its surface in SBF and that the Ag^+^ ions are slowly released into fetal bovine serum. This Ti metal exhibited a strong antibacterial effect against *Staphylococcus aureus*, as expected (Kizuki et al., 2014).

It is apparent from these results that bone-bonding bioactive Ti metal and its alloys possessing various functions can be obtained by forming Ca-deficient calcium titanate containing various ions on their surfaces by the modified alkali and heat treatment.

## Modification of the Acid and Heat Treatment

The simple acid and heat treatment described above is not effective for many of the Ti-based alloys in inducing bone-bonding bioactivity, since the alloying elements are generally hardly released during the acid treatment and are enriched on the surface of the alloys during the subsequent heat treatment. For example, Al and V of the Ti–6Al–4V alloy are hardly released during the first H_2_SO_4_/HCl acid treatment, and are segregated on the surface of the alloy during the subsequent heat treatment at 600°C. Consequently, the Ti–6Al–4V alloy subjected to the simple acid and heat treatment does not form the apatite on its surface in SBF (Yamaguchi et al., 2015).

On the other hand, the alloying elements Zr, Nb, and Ta are generally more or less selectively released from the surface of the Ti-based alloys during the NaOH treatment. Therefore, if the Ti-based alloys are subjected to the acid and heat treatment after the NaOH treatment, they form titanium oxide on their surfaces to become able to form the apatite on their surfaces in SBF and the body environment. For example, if the Ti–15Zr–4Nb–4T alloy is soaked in 0.5 or 50 mM HCl solution at 40°C for 24 h after the NaOH treatment and then subjected to heat treatment at 600°C for 1 h, it forms anatase and rutile on its surface and exhibits apatite formation on its surface in SBF (Yamaguchi et al., 2011). The acid and heat treatment after the NaOH treatment gives stable apatite formation in SBF compared with the simple alkali and heat treatment, even in the case of pure Ti metal. Its apatite-forming ability increases with an increasing concentration of the acid solution (Pattanayak et al., 2009a, 2011a,b).

This modified acid and heat treatment as well as the simple acid and heat treatment are especially useful when they are applied to porous Ti metal.

## Application of the Acid and Heat Treatment to Porous Ti Metal

Natural bone consists of a cancellous portion and a cortical portion. The cancellous portion consists of three dimensionally connected pores. If such a porous structure is introduced into the Ti metal, its elastic modulus can be decreased down to the level of human bone, making the Ti metal mechanically compatible with the surrounding bone. In addition, the Ti metal with such a porous structure can be easily fixed to the surrounding bone by bone ingrowth into the pores.

Ti metal with a porous structure that is well controlled in terms of the size and arrangement of the pores can be fabricated by sintering with metal powder containing volatile substances (Pattanayak et al., 2009b), as well as by 3D-printing methods such as a selective melting of the metal powders with a laser or electron beam (Pattanayak et al., 2011c and Fukuda et al., 2011b).

**Figure 13:**
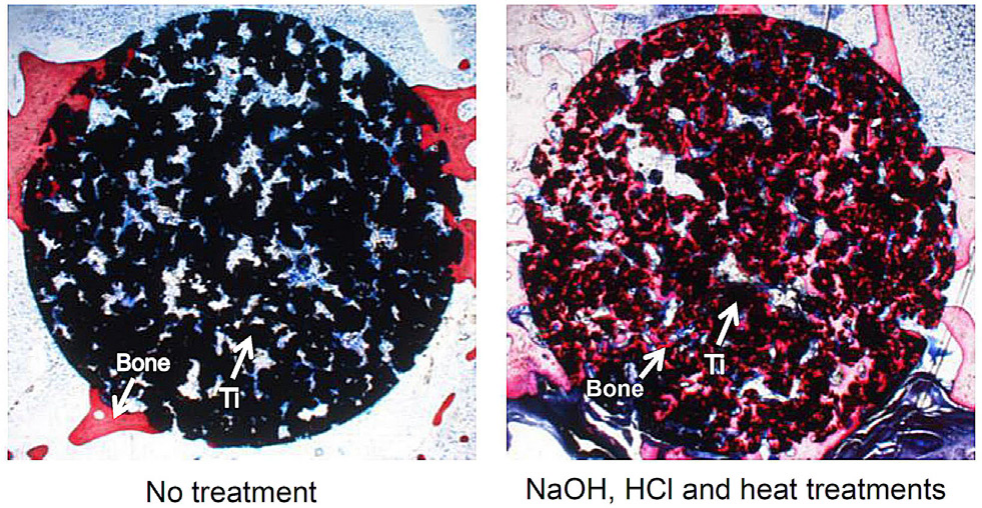
**Bone formation in porous Ti metals subjected to no treatment and NaOH, HCl, and heat treatments, 26 weeks after implantation into rabbit femur, after Takemoto et al. (2005) and Tanaka et al. (2009)**.

However, bone ingrowth into the pores is limited to only a shallow region of the porous structure for the naturally porous Ti metal, as shown in Figure 13 (Takemoto et al., 2005). In contrast, bone penetrates into a deeper region of the porous Ti metal subjected to the chemical and heat treatments for inducing the bone-bonding bioactivity described above, as shown in Figure 13 (Tanaka et al., 2009; Kawai et al., 2013).

**Figure 14:**
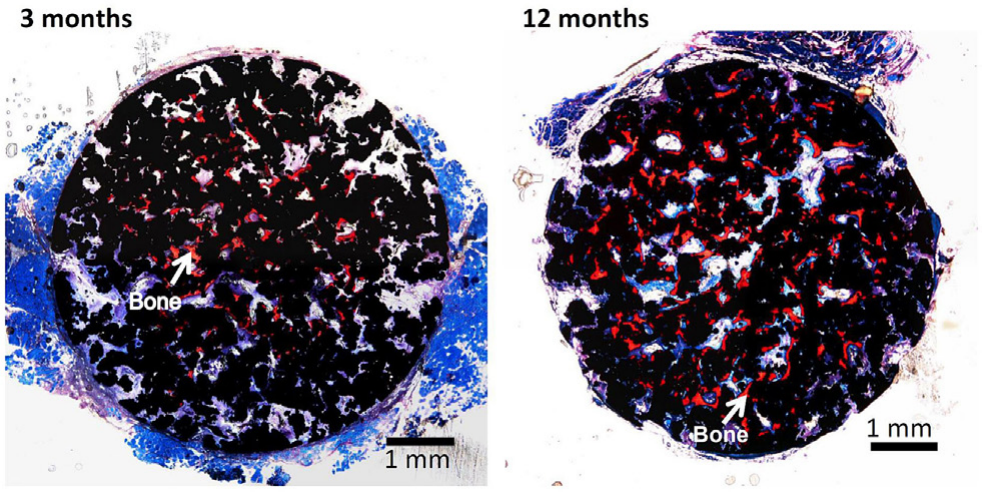
**Bone formation in porous Ti metals subjected to NaOH, HCl, and heat treatments, 3 and 12 months after implantation into muscle of beagle dog**. Reproduced from Takemoto et al. (2006) with permission Elsevier.

It should be noted here that the bioactive porous Ti metal forms bone tissue in the porous structure not only in the bone defect, but also in muscle, as shown in Figure 14 (Fujibayashi et al., 2004; Takemoto et al., 2006). The former bone formation in bone defect is called osteoconduction, whereas the latter ectopic bone formation in muscle is called osteoinduction. It is interesting to note that the bioactive porous Ti metal forming the rutile on its surface by the simple (Kawai et al., 2014) or modified acid and heat treatment exhibit active osteoinductivity, while the bioactive porous Ti metal forming the sodium titanate or Ca-deficient calcium titanate on its surface by the simple or modified alkali and heat treatment exhibit only slight or no osteoinductivity. The latter poor osteoinductivity might be attributed to the adverse effect of the released Na^+^ or Ca^2+^ ions on the activity of living cells in the narrow space of the pores by increasing the local pH in the environment.

It is expected that the osteoconductive and osteoinductive porous Ti metals prepared by the simple or modified acid and heat treatments will be used for various applications in orthopedics and dentistry.

## Clinical Applications of Osteoconductive and Oseteoinductive Porous Ti Metal Prepared by Acid and Heat Treatment

Porous Ti metals with a three-dimensional porous structure analogous to human cancellous bone are prepared by the sintering or 3D-printing of metal powders, as described above. They exhibit osteoconductivity as well as osteoinductivity when subjected to simple or modified acid and heat treatment. These porous Ti metals can be used as important bone substitutes in many applications.

**Figure 15:**
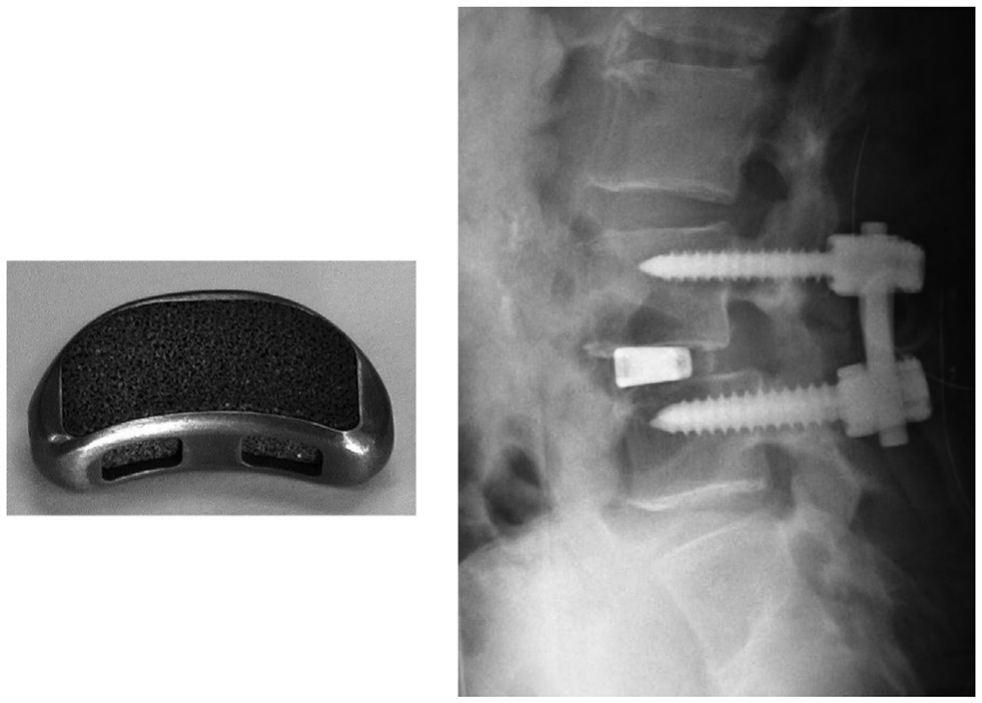
**Spinal fusion device of porous Ti metal subjected to NaOH, HCl and heat treatments (left hand side) and its clinical application (right hand side)**. Reproduced from Fujibayashi et al. (2011) with permission Springer.

For example, a porous Ti metal that is 50% in porosity and 300 μm in average pore size prepared by the sintering method was subjected to 0.5 mM HCl and heat treatment after the NaOH treatment had formed anatase and rutile on its surface. It was implanted into a canine for spinal interbody fusion (Takemoto et al., 2007). Based on this successful result, it was subjected to clinical trials as a spinal fusion device in five human patients between November 2008 and June 2009, as shown in Figure 15 (Fujibayashi et al., 2011). Conventional spinal fusion devices need an autograft for fixation to the surrounding bone, since they cannot bond to living bone, whereas the present device can be fixed without an autograft, as it does bond to living bone. All of the clinical cases to date have resulted in a successful outcome.

## Conclusion

A positively charged titanium oxide layer was grown on Ti metal, when it was heat-treated after exposure to strong acid solution, whereas negatively charged sodium titanate layer was grown on Ti metal, when it was heat-treated after exposure to strong alkali solution. Both the resultant products formed an apatite on their surfaces in a SBF and bonded to the living bone in the rabbit tibia. The latter product was successfully clinically applied to artificial hip joint.

A negatively charged calcium-deficient calcium titanate layer was formed on Ti metal and its alloys, when they were heat-treated after exposure to NaOH and CaCl_2_ solutions and finally soaked in a hot water. They also formed the apatite on their surfaces in SBF and tightly bonded to the living bone. Bone growth promoting ions such as Mg, Sr, and Zn, as well as antibacterial ions such as Ag were incorporated into the calcium titanate surface layers, in order to be slowly released in the living body.

Porous Ti metal grown with the positively charged titanium oxide on its surface exhibited not only osteoconduction but also osteoinduction. The resultant product is being subjected to clinical trials as a spinal fusion device.

This kind of novel bioactive materials exhibiting various kinds of biological functions as well as intrinsic high mechanical strength likely to play an increasingly more important role in repairing damaged bone tissues.

## Conflict of Interest Statement

The authors declare that the research was conducted in the absence of any commercial or financial relationships that could be construed as a potential conflict of interest.
